# Predictive analytics in bronchopulmonary dysplasia: past, present, and future

**DOI:** 10.3389/fped.2024.1483940

**Published:** 2024-11-20

**Authors:** Bryan G. McOmber, Alvaro G. Moreira, Kelsey Kirkman, Sebastian Acosta, Craig Rusin, Binoy Shivanna

**Affiliations:** ^1^ Division of Neonatology, Department of Pediatrics, University Hospital, University of Texas Health Science Center at San Antonio, San Antonio, TX, United States; ^2^ Division of Neonatology, Department of Pediatrics, Texas Children’s Hospital, Baylor College of Medicine, Houston, TX, United States; ^3^ Division of Pediatric Cardiology, Department of Pediatrics, Texas Children’s Hospital, Baylor College of Medicine, Houston, TX, United States

**Keywords:** bronchopulmonary dysplasia, predictive analytics, artificial intelligence, machine learning, personalized medicine, bioinformatics

## Abstract

Bronchopulmonary dysplasia (BPD) remains a significant complication of prematurity, impacting approximately 18,000 infants annually in the United States. Advances in neonatal care have not reduced BPD, and its management is challenged by the rising survival of extremely premature infants and the variability in clinical practices. Leveraging statistical and machine learning techniques, predictive analytics can enhance BPD management by utilizing large clinical datasets to predict individual patient outcomes. This review explores the foundations and applications of predictive analytics in the context of BPD, examining commonly used data sources, modeling techniques, and metrics for model evaluation. We also highlight bioinformatics’ potential role in understanding BPD's molecular basis and discuss case studies demonstrating the use of machine learning models for risk prediction and prognosis in neonates. Challenges such as data bias, model complexity, and ethical considerations are outlined, along with strategies to address these issues. Future directions for advancing the integration of predictive analytics into clinical practice include improving model interpretability, expanding data sharing and interoperability, and aligning predictive models with precision medicine goals. By overcoming current challenges, predictive analytics holds promise for transforming neonatal care and providing personalized interventions for infants at risk of BPD.

## 1 Introduction

Bronchopulmonary dysplasia (BPD) is a chronic lung disease primarily caused by inflammation and lung injury due to mechanical ventilation and supplemental oxygen therapy (1). This condition disrupts the growth and development of alveoli and pulmonary vasculature and plays a significant role in the prognosis and long-term outcomes of premature neonates (2). Despite advances in neonatal care and improved survival of premature infants, BPD rates remain high, affecting as many as 18,000 infants annually in the US (1). Definitions of BPD have evolved since it was first described in 1967, with the National Institute of Child Health and Human Development (NICHD) and the Neonatal Research Network (NRN) adopting severity-based grading systems based on respiratory support at 36 weeks postmenstrual age (PMA) (3, 4). Some studies suggest defining BPD at 40 weeks PMA may better predict serious long-term respiratory outcomes (5).

Managing BPD remains challenging due to the rising survival rates of extremely premature infants and variability in clinical practices. Current strategies include non-invasive ventilation, surfactant therapy, and early caffeine administration (6). Although postnatal steroid therapy aids in weaning neonates from mechanical ventilation, studies have not demonstrated a significant reduction in overall BPD rates (7). Individualized care is crucial given the multifactorial nature of BPD, with factors such as oxygen therapy, ventilation methods, medications, nutrition, and genetics influencing outcomes. Predictive analytics holds promise for developing more targeted and personalized therapies to improve the management and prognosis of BPD.

## 2 Foundations and techniques of predictive analytics

Predictive analytics in healthcare encompasses a variety of statistical and machine learning techniques aimed to forecast predictions about future outcomes based on historical data (8, 9). The central idea is to utilize past clinical trajectories of large cohorts to predict outcomes for patients with similar clinical characteristics. In this regard, supervised machine learning techniques are commonly used for predictive modeling, where algorithms are trained on labeled datasets to understand the relationship between input features (e.g., clinical data) and known outcomes. In contrast, unsupervised machine learning is used to discover patterns or clusters within data without predefined labels, offering insights into developing clinical phenotypes (8, 10).

### 2.1 Commonly used data sources and types in predictive modeling

Predictive modeling leverages multi-modal data sources to enhance clinical outcomes and optimize care strategies (Table 1). Primary data sources are from electronic health records (EHRs) (9), which provide comprehensive, longitudinal data on patient demographics, medical history, treatment plans, and clinical outcomes. Imaging data, such as ultrasounds and MRI scans (11, 12), also play a critical role in diagnosing and predicting the progression of various disorders. Additionally, real-time monitoring data, capturing vital signs such as heart rate, respiratory rate, and oxygen saturation, are increasingly being integrated into predictive models (13–15). This high frequency, time-series data is instrumental in the early detection of conditions like sepsis or respiratory distress. Other valuable data sources include genomic data and other bioinformatics, which can provide insights into congenital conditions and inform personalized treatment plans.

**Table 1:** Key components of predictive analytics in BPD management.

Component	Description	Examples
Data sources	Integration of clinical, imaging, real-time monitoring, and genomic data to enhance prediction accuracy.	EHRs, MRIs, lung ultrasounds, vital signs, and genomic datasets
Machine learning techniques	Advanced algorithms for identifying patterns and predicting outcomes in neonates at risk of BPD.	Logistic regression, random forests, convolutional neural networks (CNNs), recurrent neural networks (RNNs), multi-layer perceptrons (MLPs)
Model evaluation metrics	Metrics used to evaluate predictive performance, such as accuracy, sensitivity, specificity, and AUC.	AUC > 70.0 preferred for clinical use; balanced sensitivity and specificity
Applications	Predicts BPD risk and severity, enables early interventions, and guides personalized care.	Japan GBDT model; Korean two-stage MLP model
Challenges	Barriers include data fragmentation, overfitting, lack of model interpretability, and bias in predictions.	Standardized data sharing needed, explainable AI to ensure model transparency
Opportunities	Enhancing precision medicine and encouraging multi-institutional collaboration through predictive models.	Early interventions using respiratory monitoring, integration of genomic data for personalized care

BPD, bronchopulmonary dysplasia; EHR, electronic health record; MRI, magnetic resonance imaging; AUC-ROC, area under the receiving operator curve; GBDT, gradient boosting decision tree; AI, artificial intelligence.

### 2.2 Techniques and algorithms for predictive analytics

Multiple methods can be utilized to develop predictive algorithms in healthcare. A common approach involves defining a time period before the event is predicted (usually called the pre-deterioration class) and identifying a time period where no event occurs (the control class) (13). Standard classification algorithms can then be applied to distinguish between these two classes, resulting in an algorithm capable of detecting the pre-deterioration state that occurs before the event of interest.

Linear regression, logistic regression, and Cox proportional hazards models are commonly used as a first step (10). For larger and more complicated datasets, more sophisticated machine learning approaches, such as decision trees, random forests, and support vector machines, offer enhanced robustness and accuracy in predictions (8, 9). Deep learning, a subset of neural network machine learning, has also gained prominence due to its ability to model intricate patterns in data through neural networks (16–18). Convolutional neural networks (CNNs) are particularly effective in image analysis (11, 12, 19), while recurrent neural networks (RNNs) are particularly effective in handling time-series data (20). Additionally, ensemble methods, which combine multiple algorithms to improve predictive performance, are also frequently utilized (17). The choice of predictive model depends on the amount and complexity of data, with more advanced models typically required for larger and more intricate datasets.

### 2.3 Evaluation metrics for predictive models

Evaluating the performance of predictive models is critical to ensuring their reliability and efficacy in clinical settings. Common evaluation metrics include alert rate, accuracy, sensitivity, specificity, precision, and the area under the receiver operating characteristic curve (AUC-ROC) (13, 17, 18). Accuracy measures the proportion of correct predictions out of the total predictions, providing a general sense of model performance. Sensitivity (or recall) assesses the model's ability to identify true positive clinical events correctly, to ensure that at-risk patients are accurately detected. Specificity evaluates the model's ability to identify true negatives correctly, preventing the misdiagnosis of healthy individuals. Precision, which is the proportion of true positives among the predicted positives, reflects the model's reliability in identifying true positive predictions.

The AUC-ROC provides a comprehensive measure of model performance across different threshold settings, balancing sensitivity and specificity to give an overall picture of the model's discriminatory ability. In clinical settings, an AUC value above 0.70–0.75 is generally considered acceptable, while values above 0.80–0.85 are preferred, indicating a strong ability to differentiate between patients who will and will not experience the outcome. Alert rate is the number of alerts that are generated per patient per day, which is important to understand from a clinical workload management perspective (i.e., you can have high performance, but if it comes at the cost of hundreds of alarms per patient per day, the model is not clinically viable). These metrics offer a robust framework for assessing and validating predictive models, ensuring their clinical utility and effectiveness.

**Figure 1:**
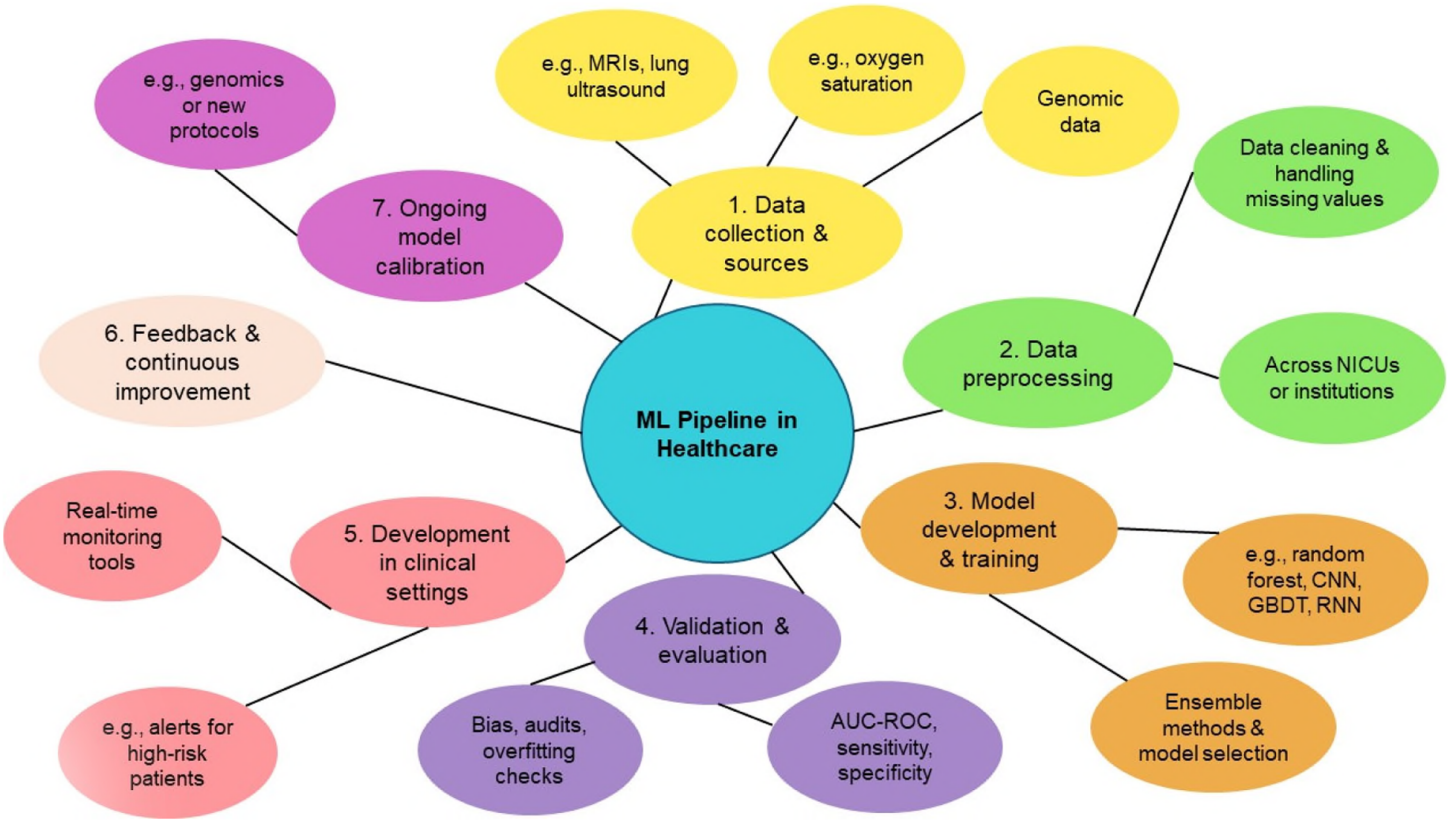
Overview of the machine learning (ML) pipeline in healthcare, illustrating the key stages from data collection to model deployment and continuous improvement. The process begins with Data Collection and Sources (Step 1), including clinical data such as MRIs, genomics, and oxygen saturation metrics. Data Preprocessing (Step 2) involves cleaning datasets and addressing missing values across healthcare institutions. In Model Development and Training (Step 3), algorithms like Random Forests, CNNs, RNNs, and gradient boosting decision trees (GBDTs) are utilized, with ensemble methods aiding in model selection. Validation and Evaluation (Step 4) ensures performance reliability through metrics such as AUC, sensitivity, and specificity, alongside bias audits. Deployment in Clinical Settings (Step 5) introduces real-time monitoring tools and alerts for high-risk patients. Feedback and Continuous Improvement (Step 6) and Ongoing Model Calibration (Step 7) ensure sustained performance and adaptability in evolving clinical environments.

Performance metrics are calculated and reported on both a training data set and a separate independent data set for validation purposes. Importantly, the validation data set is not used in the initial training of the model. Validation testing is required to ensure that these machine learning models are not overfitting the training data. Model overfitting occurs when the training process of the algorithm essentially allows the model to memorize the inputs and outputs for a given data set. This can occur if there are a large number of input variables (also called model input features) and a low number of observations. A model that performs well on both training data and independent validation data sets at the same time is considered to have good generalization, which is critical to a successful clinical deployment. A workflow for predictive analytics in healthcare can be seen in Figure 1.

## 3 Predictive modeling and bioinformatics

### 3.1 Predictive models for risk stratification and prognosis

Previous studies have attempted to develop functional predictive models for BPD, with varied results (Table 2) (49–51). These models usually consisted of formulas or web calculators based on demographic factors such as birth weight, gestational age, and sex. While many of these studies described a high predictive performance, they were often inconsistent with one another for various reasons. Among these were differences in their definitions of BPD, such as the outcome, small sample sizes, and poor handling of missing data. Of note, a recent meta-analysis of seven studies using lung ultrasound scores in neonates to predict BPD showed significant diagnostic accuracy in predicting BPD at 7 & 14 days of life (52).

**Table 2:** Examples of predictive modeling approaches for BPD.

Study	Outcome	Timing of model assessment	Analysis sample size	C-Statistic	External validation?
Chioma 2024 (21)	BPD/death (2018 definition)	D7	99	Echocardiographic data + clinical data 0.98 [0.96–1.00]	No
Chou 2024 (22)	BPD (2018 definition)	Before 24h	480	Chest radiography	No
Gao 2023 (23)	Mild/moderate/severe BPD (2001 definition)	Unclear	237	Clinical data 0.9051	Yes
Kostekci 2023 (24)	Moderate/severe BPD/death (2001 definition)	D1, D7, D14, D28	124	Clinical data day 1–0.564–0.797 day 7–0.751–0.935 day 14–0.791–0.886 day 28–0.791–0.881	No
Moreira 2023 (25)	BPD (2001 definition)	D5	97	Blood biomarkers 0.961 [0.897–1.00]	No
Ou 2023 (26)	Mild/moderate/severe BPD (2001 definition)	D7	102	Clinical data + blood biomarkers 0.96 [0.90–1.00]	No
Shen 2023 (27)	Mild/moderate/severe BPD (2001 definition)	D7, D14	542	Clinical data 0.925 [0.902–0.948]	No
Ahmed 2022 (28)	Moderate/severe BPD (supplemental O_2_ at 36w)	Before 72h	42	Urinary proteomics 2 protein panel—0.92 [0.84–1.00] 3 protein panel—0.94 [0.86–1.00]	No
Alonso-Ojembarrena 2022 (29)	Moderate/severe BPD (any respiratory support at 36w)	D1, D3, D7, D14	133	LUS score + blood biomarkers + clinical data day 1–0.41 [0.33;0.50] day 3–0.52 [0.43;0.61] day 7–0.83 [0.75;0.89] day 14–0.85 [0.77;0.90]	Yes
Bhattacharjee 2022 (30)	Severe BPD (>30% O_2_ or PPV/CPAP at 36w or discharge)	D3	64	RSS score day 3–0.61 [0.47;0.75]	Yes
Greenberg 2022 (31)	Grades 1–3 BPD/death (2019 definition)	D1, D3, D7, D14, D28	9,181	Clinical data Range from 0.629 to 0.741 depending on DOL and variable used for prediction	No
Kindt 2022 (32)	Moderate/severe BPD (2001 definition)	First week	52	Plasma proteomics + clinical data 3 protein panel + GA—0.87	No
Umapathi 2022 (33)	Moderate/severe BPD/death (supplemental O_2_ at 36w)	First week	98	Echocardiographic data + GA 0.97 [0.93;0.99]	No
Zayat 2022 (34)	Moderate/severe BPD (supplemental O_2_ or respiratory support at 36w)	D14	3662	Clinical data median of 10 datasets 0.82, range 0.819–0,823	No
Aldecoa-Bilbao 2021 (35)	Moderate/severe BPD (supplemental O_2_ or PPV at 36w)	D7	89	LUS score + clinical data day 7–0.80 [0.70;0.90]	Yes
Alonso-Ojembarrena 2021 (36)	BPD 36w (Walsh test^a^)	D0, D3, D7, D14	298	Modified LUS score + clinical data day 3–0.77 [0.68;0.85] day 7–0.80 [0.74;0.85] day 14–0.77 [0.69;0.83]	Yes
Baud 2021 (37)	Moderate/severe BPD/death (respiratory support at 36w, Walsh test if FiO_2_ 22–29%)	At birth	523	Clinical data 0.73 [0.68;0.77]	Yes
Dai 2021 (38)	Severe BPD or death due to respiratory insufficiency (NICHD definition)	Unclear	245	Genetics + clinical data 0.915 [0.843;0.987]	No
Gerull 2021 (39)	BPD/death at 36w	D7	229	Clinical data + blood biomarkers 0.86 [0.76;0.95]	Yes
Khurshid 2021 (40)	BPD/death (supplemental O­_2_ or PPV at 36w)	D1, D7, D14	9,006 (day 1) to 3,899 (day 14)	Clinical data Range of model performance in infants <33w GA day 1–0.811–0.862 day 7–0.812–0.886 day 14–0.815–0.884 Range of model performance in infants <29w GA day 1–0.699–0.782 day 7–0.706–0.783 day 14–0.708–0.790	No
Liu 2021 (41)	BPD (2019 definition)	D1, D2, D3, D6, D9, D12	130	LUS score + clinical data day 1–0.65 [0.56;0.74] day 2–0.72 [0.64;0.80] day 3–0.73 [0.65;0.81] day 6–0.77 [0.69;0.84] day 9–0.84 [0.76;0.90] day 12–0.83 [0.76;0.89]	Yes
Mohamed 2021 (42)	BPD (supplemental O_2_ or respiratory support at 36w)	D3, D7, D14	152	LUS score + clinical data day 3–0.96 [0.94;0.99] day 7–0.97 [0.94;0.99] day 14–0.95 [0.92;0.98]	Yes
Shim 2021 (43)	Moderate/severe BPD (NIH definition)	D0	4,600	Clinical data 0.84 [0.84;0.84] (BPD only) 0.78 [0.78;0.79] (BPD/death)	Yes
Song 2021 (44)	Moderate/severe BPD/death (NIH definition)	D7	556	Clinical data + blood biomarkers 0.861 [0.819;0.903]	No
Soullane 2021 (45)	BPD (supplemental O_2_ or respiratory support at 36w or at discharge)	D10	191	Clinical data 0.76	No
Ushida 2021 (46)	BPD (supplemental O_2_ at 36w)	D0	20,771	Clinical data 0.84 [0.84;0.85] (BPD) 0.85 [0.85;0.86] (BPD/death)	Yes
Woods 2021 (47)	Moderate/severe BPD (respiratory support at 36w)	D1, D3, D7	96	LUS score + clinical data day 1–0.56 [0.44;0.67] (moderate/severe BPD) or 0.59 [0.47;0.70] (grade II/III BPD) day 3–4–0.64 [0.52;0.74] or 0.77[0.66;0.85] day 7–0.61 [0.49;0.72] or 0.67 [0.55;0.77]	Yes
Zhang 2021 (48)	BPD (2019 definition)	D3, D7, D14	414	Clinical data day 3–0.832 day 7–0.876 day 14–0.880	No

D, day of life; LUS, lung ultrasound; PPV, positive pressure ventilation.

^a^ Walsh test: oxygen reduction test to determine oxygen dependency in infants at 36w; if baby can maintain SpO_2_ at >90% for 30 min under room air conditions, they are considered not to have BPD.

In 2022, the National Institutes for Child Health and Development updated a previous model for calculating BPD in infants with gestational ages between 23 and 28 weeks (31, 53). This estimator now includes factors such as antenatal steroid administration and the occurrence of surgical necrotizing enterocolitis. Unfortunately, these predictive models do not yet incorporate omics data, as this study area is still under research. However, integrating omics data could significantly enhance the predictive power of these models.

In recent years, researchers have begun using artificial intelligence (AI) or high computational analyses to decipher large data (54, 55). These AI models serve as rapid tools that can be leveraged for predictive modeling that can optimize accuracy. This capability could also be applied to precision medicine on a patient-by-patient basis. For example, an AI-powered predictive model integrated into a hospital's electronic health system could quickly and affordably assess an individual patient's genome, vital sign capture, or mechanical ventilator data for BPD risk factors, offering real-time decision support based on personalized risk factors.

### 3.2 Bioinformatics

Bioinformatics plays a significant role in understanding the molecular mechanisms underpinning BPD. Omics could participate in precision medicine in two ways: prediction and treatment. The integration of omics data enables the development of predictive models that can identify preterm infants at higher risk for BPD. By using genomic changes observed in the first few days of life, researchers can categorize neonates based on their risk profiles. This stratification can help during clinical trial selection by recruiting neonates at high risk for BPD, making sure that treatments are tested on individuals most likely to benefit. In addition, with a better understanding of the dysregulated pathways, new therapies can be developed to specifically target modifiable mechanisms, aiding in the development of new therapies. The following are examples of bioinformatic studies used to better understand BPD:

• Transcriptomics: Genes involved in ferroptosis and T-cell immunity have been associated with BPD (25, 56).
• Metabolomic: Pathways, including the citrate cycle, alanine, aspartate, and glutamate metabolism have been linked with BPD (57).
• Microbiomics: Increased Proteobacteria and decreased Firmicutes in the gut modulate systemic inflammatory levels of IL-1β, IL-6, and TNF-α, impacting lung health (58).

## 4 Case-Based example of using predictive analytics in BPD

Two recent studies illustrate the practical application of ML models in predicting BPD risk and severity.

### 4.1 Gradient boosting model for early BPD prediction

This study presented the application of machine learning, specifically gradient boosting decision trees (GBDT), to predict the immediate postnatal risk of death or BPD in very preterm and very low birth weight infants using data from a nationwide Japanese database (59). The GBDT algorithm, trained on clinical variables available at birth (e.g., gestational age, birth weight, Apgar scores, and maternal factors), accurately identified infants at high risk for adverse outcomes, enabling for potential early intervention strategies.

The clinical implications of this study are significant, as early and accurate risk stratification can be a powerful tool for clinicians. The ability to predict death or BPD shortly after birth enables healthcare providers to better allocate resources, such as assigning high-risk infants to specialized teams, tailoring respiratory support, and optimizing nutritional strategies. Moreover, the GBDT model's interpretability aids clinicians in identifying key risk factors, enhancing decision-making and parental counseling. This level of personalized risk assessment also aligns with the push towards precision medicine in neonatology, where interventions can be targeted to those who need them the most. Implementing such predictive models in clinical practice could improve outcomes by enabling earlier interventions for infants at risk of BPD or death. However, successful integration requires model validation across diverse populations beyond Japan, and seamless implementation into clinical workflows with proper staff training.

### 4.2 Two-stage neural network model for BPD severity prediction

Using a two-stage machine learning approach, Hwang et al. took data from a nationwide cohort of VLBWs to build predictive models that help in the early identification of those at high risk of developing BPD (60). The first stage involved training models to predict moderate to severe BPD, and the second stage focused on predicting the presence and severity of BPD based on new clinical data gathered later in the neonatal period. By incorporating longitudinal data, the model aimed to enhance prediction accuracy by reflecting changes in the infant's condition over time.

The study used clinical and demographic data to create its prediction model. They included gestational age, birth weight, respiratory support requirements, and other indicators known to influence the risk of BPD in preterm infants. The results demonstrated that the two-stage learning-based approach could outperform traditional single-stage models, achieving higher predictive accuracy and identifying at-risk infants with good sensitivity and specificity.

This study's clinical relevance lies in early BPD prediction, enabling targeted interventions and resource allocation in NICUs. The model helps clinicians identify infants for preventive strategies like optimized ventilation and early treatments. Its two-stage approach mirrors real-world decision-making, making it adaptable and valuable in dynamic NICU settings, ultimately improving patient outcomes with personalized care.

## 5 Challenges and limitations of predictive analytics

Predictive modeling in healthcare holds significant promise; however, various challenges and limitations can affect the accuracy and reliability of these models.

### 5.1 Data gaps

#### 5.1.1 Incomplete and unstandardized data

Missing data, often resulting from incomplete records or data entry errors, can introduce bias into model predictions (61–65), reducing its accuracy and reliability. Further, the data may vary greatly in size, form, and format; they are often complex, heterogeneous, poorly annotated, and frequently unstructured, preventing effective modeling (66).

#### 5.1.2 Data collection bias

Certain populations may be underrepresented in datasets, causing biased predictions that fail to generalize effectively across diverse patient groups (67). As a result, the trained model may perform poorly for these underrepresented populations.

#### 5.1.3 Infrequent updates

Medical records or patient data may be updated inconsistently. If a model is trained on outdated information, it might not accurately capture current trends or changes in patient conditions.

### 5.2 Real-Time data integration challenges

#### 5.2.1 Heterogeneous data

Heterogeneous data poses another challenge; variations in data entry practices, the use of different types of data sources (e.g., EHRs, wearable devices, and medical imaging), and incompatible formats and standards from different healthcare systems can hinder the seamless exchange and integration of data (64, 65, 68–70).

#### 5.2.2 High volume and high velocity data

The high volume of data can overwhelm traditional data processing systems, while the high velocity of real-time data from wearable devices and monitoring systems demands rapid processing and analysis to maintain clinical relevance (70).

#### 5.2.3 Delay in data availability

The laboratory results or diagnostic imaging reports may experience processing delays, hindering the ability to make accurate real-time predictions.

#### 5.2.4 Constantly changing data

Additionally, the dynamic nature of healthcare data introduces further complexity; as healthcare knowledge and practices evolve, maintaining up-to-date models becomes challenging (71). Moreover, patient health conditions can also change rapidly, requiring models to adapt in real time (71, 72).

#### 5.2.5 Heterogeneous data source platforms

Often, the data source platforms used by the institutions vary, creating a barrier for effective real-time data integration (73).

#### 5.2.6 Data privacy and security

Real-time integration of data involves handling sensitive healthcare data, which raises concerns about privacy and security (74). Strict compliance with regulations like the US Health Insurance Portability and Accountability Act (HIPAA) can complicate data usage, sharing, and integration. Ensuring compliance with regulations like HIPAA is essential to mitigate risks of unauthorized access or breaches of sensitive data. Institutions must also incorporate legal safeguards that align with emerging global frameworks, such as the EU's General Data Protection Regulation (GDPR), which emphasizes data minimization and user control.

### 5.3 Model complexity and interpretability

While predictive modeling has a significant impact on healthcare, the complexity and interpretability of these models pose challenges and limitations. Advanced predictive models, particularly those built with AI and ML, often result in “black-boxes” with intricate internal workings that are not easily interpretable by clinicians (75). This lack of clarity raises concerns about trust, as it becomes difficult to understand or validate how these models generate predictions. As a result, healthcare professionals may hesitate to adopt these tools at the bedside, fearing errors, misjudgments, or liability issues without being able to challenge or explain the recommendations.

The interpretability problem becomes especially critical in high-stakes environments, such as diagnosing life-threatening conditions or determining optimal treatment plans (76). A predictive model's lack of transparency can undermine clinician confidence, especially when it suggests withholding treatment or recommending experimental interventions. This uncertainty affects decision-making and complicates patient consent, as families may be uneasy trusting decisions without clear human explanations.

Another challenge with these complex models is the risk of overfitting, where a model learns patterns from training data too precisely, including noise or irrelevant correlations (77). While this may produce highly accurate predictions on known datasets, the model's performance may worsen when applied to new, real-world data. This poor generalization can lead to biased predictions, especially if the training data does not represent the diversity of patient populations adequately. For example, a model trained primarily on data from urban hospitals might struggle to provide accurate predictions for rural or underserved communities.

Ensuring robustness and reliability in predictive models also requires balancing complexity with simplicity. While deep learning models may offer superior predictive power, simpler algorithms—such as logistic regression or decision trees—might be more suitable in clinical settings because they are easier to interpret. Explainability plays a crucial role in integrating predictive analytics into healthcare.

### 5.4 Resource, cost constraints, disparities in care, and regulatory and ethical concerns

Predictive analytics in healthcare introduces various ethical and regulatory challenges, including patient consent, data privacy, and the potential misuse of sensitive information. As noted by Carini and Seyhan, the effectiveness of predictive models relies on access to high-quality data, raising questions about data ownership, security, and interoperability (78). Institutions must invest in infrastructure that supports ethical AI development while managing the growing burden of regulatory obligations. Developing, implementing, and maintaining predictive analytics tools is resource- and cost-intensive, demanding significant investment in technology, data infrastructure, and skilled personnel.

Beyond initial implementation, organizations must budget for the continuous upkeep of these systems, which involves periodic recalibration of algorithms to address evolving patient demographics and medical practices. Furthermore, as Marques et al. emphasize, smaller healthcare providers or facilities in underserved areas may struggle to afford these advanced technologies, potentially widening disparities in access to care (79). Additionally, the integration of predictive analytics requires robust training programs to equip healthcare professionals with the necessary skills to interpret and utilize AI-generated recommendations effectively. Institutions must also account for potential disruptions and interoperability issues when incorporating these tools into existing workflows, as outdated or fragmented systems may impede seamless adoption.

There are also hidden costs associated with data management. Predictive analytics depends on large, well-annotated datasets, which require significant storage capacity and security protocols. Ensuring compliance with privacy regulations such as the GDPR adds another layer of complexity, as it may necessitate specialized software for data anonymization and consent management. Moreover, these tools often rely on cloud-based infrastructure, incurring recurring costs for data hosting and cybersecurity measures to prevent breaches.

Finally, the high initial and operational costs may lead institutions to rely heavily on third-party vendors, raising additional concerns about dependency and data sovereignty. Over-reliance on external partners can also complicate accountability in the event of errors or data breaches, further increasing the burden on institutions to establish clear governance frameworks that align with ethical standards and regulatory requirements.

### 5.5 Data bias and misuse risks

Predictive models, though powerful, can unintentionally perpetuate biases if not carefully designed. Marques et al. emphasize that AI models are only as reliable as the data they are trained on, and these datasets often reflect existing social biases (79). This can lead to inequitable care or discrimination, particularly against marginalized populations. To address these concerns, predictive systems must undergo regular auditing to detect bias, and the development process should include input from diverse stakeholders to ensure fair outcomes.

Another emerging concern is the potential misuse of predictive tools, particularly when these models are deployed without appropriate oversight. In some cases, predictive analytics could be misused to ration care, denying necessary services to patients deemed “low risk” by an algorithm. Regulatory frameworks must ensure that predictive tools are not only effective but also used ethically, with clear accountability mechanisms to prevent misuse. Transparency is essential—healthcare providers and patients should have access to detailed explanations of how predictions are generated, as well as the ability to contest decisions based on algorithmic outputs.

Finally, mitigating risks requires a culture of accountability in AI development, with governance frameworks for bias assessments and addressing unintended consequences. Engaging diverse stakeholders throughout the AI lifecycle ensures fairness and equity, improving model reliability and fostering trust among patients and providers for more just, effective healthcare.

### 5.6 Accountability and trust in predictive systems

Predictive analytics in healthcare complicates accountability, especially when algorithm-driven decisions cause harm. The lack of transparency makes it difficult to assign responsibility, posing challenges for both developers and healthcare providers (78). Trust in predictive systems requires transparency, ongoing validation, and explainable AI to help clinicians understand outputs. Clear ethical guidelines outlining stakeholder responsibilities are essential for public trust and safe technology integration.

### 5.7 Challenges and limitations of BPD predictive models

Despite the presence of many BPD predictive models (25, 50, 51, 80, 81), only a few (31, 53) are widely available to clinicians, and practically none are used regularly in clinical practice (50). Most models have methodological flaws (small sample size, poor calibration and validation, and increased bias) and lack dynamism (50, 51). Further, most models lack comprehensive, continuous, high-definition physiological data and a mechanistic biomarker panel. Finally, the data gaps and real-time data integration challenges discussed above significantly impact BPD predictive models.

Several of the limitations discussed in this section also pose significant challenges in translating research-based predictive models into clinical practice. The next section offers strategies to address these challenges.

## 6 Future directions and opportunities

### 6.1 Enhancing model interpretability

A key priority for advancing predictive analytics is improving AI model interpretability. Complex algorithms like deep learning, despite their predictive power, face limited clinical adoption due to their “black-box” nature. Clinicians hesitate to trust opaque recommendations, especially in high-stakes areas like neonatal care. Explainable AI (XAI) frameworks are crucial to addressing this challenge (75). These frameworks aim to provide transparency by breaking down how predictions are generated, offering clinicians meaningful insights into which variables contributed to the outcome. Additionally, human-in-the-loop systems, where clinicians interact with the model to refine its predictions, can further improve adoption and create feedback loops that enhance the model's performance over time. Efforts to integrate visualization tools into clinical workflows, where model outputs are presented in intuitive dashboards, will also empower healthcare providers to use predictive analytics with greater confidence.

### 6.2 Improving data sharing and interoperability

A second key priority is improving data sharing and interoperability across healthcare institutions. Fragmented data sources, ranging from clinical records and genetic data to wearable sensor outputs—limit the effectiveness of predictive models. Interoperable systems that standardize how data is collected, stored, and exchanged will make it easier for researchers and clinicians to build robust, generalizable models. Developing shared data formats and platforms, such as Fast Healthcare Interoperability Resources (FHIR), is a step toward seamless data exchange across hospitals and research institutions (80). Large-scale collaborations supported by secure data-sharing agreements will enable the aggregation of more comprehensive datasets, increasing the statistical power of predictive models (81). However, these efforts must also navigate regulatory challenges, ensuring compliance with frameworks such as HIPAA and GDPR while maintaining the privacy and security of patient data. Institutions will need to invest in governance structures and advanced encryption technologies to enable responsible and compliant data sharing.

### 6.3 Expanding access and addressing bias

Equitable access to predictive analytics is a critical priority for the future. AI-driven models, if not carefully designed, risk exacerbating existing disparities by benefiting only well-resourced healthcare settings. Institutions in rural areas or those serving low-income populations often lack the infrastructure to adopt these advanced technologies, which may leave their patients underserved. It is crucial to ensure that predictive models are validated across diverse patient populations to prevent bias and ensure fair outcomes. This involves expanding the datasets used to train these models to include underrepresented groups, such as racial minorities and economically disadvantaged populations, who are often excluded from medical research. Regular audits for algorithmic bias must become a standard practice, with adjustments made to correct any disparities in performance. Additionally, stakeholder engagement—bringing in perspectives from underrepresented communities and clinicians working in underserved areas—will ensure the models align with the needs of all patient populations. Future policies should also focus on ensuring that these tools remain accessible and affordable, preventing cost from becoming a barrier to equitable healthcare delivery.

### 6.4 Workforce training and interdisciplinary collaboration

A skilled workforce is vital for integrating predictive analytics in healthcare. Interdisciplinary collaboration among clinicians, data scientists, engineers, and informaticians is key. Clinicians must develop basic competencies in data science and AI to actively engage with predictive tools and interpret model outputs effectively (82). This will ensure that clinical decisions informed by predictive analytics are made thoughtfully and responsibly. At the same time, data scientists and engineers need to develop a deeper understanding of clinical contexts, such as the unique challenges faced in neonatal care, to design models that align with clinical realities. Interdisciplinary training programs that bridge these gaps will foster collaboration and innovation, helping professionals from different fields work together seamlessly. Cross-disciplinary programs, like joint fellowships or certification courses, can equip future healthcare professionals and data scientists to effectively implement and advance predictive technologies.

Developing leadership in clinical informatics will also be essential to manage the intersection of technology and clinical practice (83). Clinical informaticians are key to integrating predictive models into EHRs and creating actionable decision-support tools. By designing workflows that incorporate AI predictions, they help clinicians make faster, informed decisions without being overwhelmed by data. Institutions fostering multidisciplinary collaboration will better harness these technologies to improve patient outcomes.

### 6.5 Aligning predictive analytics with precision medicine

The ultimate goal of predictive analytics in neonatal care and other fields is to advance precision medicine, where treatment plans are tailored to the individual needs of each patient. Predictive models can identify patient-specific risk factors and predict disease trajectories based on genetic, environmental, and clinical data (84). For example, models that integrate genomic data with real-time monitoring can identify biomarkers linked to early signs of respiratory complications, guiding clinicians toward more targeted interventions to reduce the severity or incidence of conditions like BPD. As Kim et al. suggest, combining genomics with continuous physiological data can yield new insights and improve individualized care strategies (83).

However, to fully harness predictive analytics in precision medicine, large, well-annotated datasets representing diverse populations are essential. Investments in data collection initiatives like biobanks and healthcare registries are crucial. Ethical considerations such as fairness, transparency, and patient autonomy must guide model deployment, with clear communication and informed consent to maintain trust. Policies governing ethical use will ensure these innovations benefit all, not just a select few.

## 7 Conclusions

The integration of predictive analytics into the management of BPD represents a transformative shift towards more personalized and precise neonatal care. By leveraging advanced machine learning techniques and incorporating diverse data sources such as EHRs, imaging, and bioinformatics, predictive models have the potential to significantly enhance risk stratification, tailor interventions, and ultimately improve outcomes for premature infants. Despite notable advancements, challenges remain, including data integration issues, model interpretability, and ethical considerations surrounding privacy and equity. Addressing these challenges through interdisciplinary collaboration, among data scientists, neonatologists, and ethicists, alongside robust validation, and ongoing research, is essential for realizing the full potential of predictive analytics in BPD management. Moving forward, a concerted effort to refine predictive models, integrate real-time data, and adhere to ethical standards will be crucial in advancing precision medicine and delivering better, individualized care for neonates at risk of BPD.
